# Multi-Informant Assessment of Adolescents’ Social–Emotional Skills: Patterns of Agreement and Discrepancy among Teachers, Parents, and Students

**DOI:** 10.3390/bs12030062

**Published:** 2022-02-25

**Authors:** María J. Mudarra, Beatriz Álvarez-González, Berta García-Salguero, Stephen N. Elliott

**Affiliations:** 1 Faculty of Education, Universidad Nacional de Educación a Distancia (UNED), 28040 Madrid, Spain; balvarez@edu.uned.es (B.Á.-G.); bgarcia@edu.uned.es (B.G.-S.); 2 Sanford School of Social and Family Dynamics, Arizona State University, Tempe, AZ 308K, USA; steve_elliott@asu.edu

**Keywords:** multi-informant assessment, social–emotional skills, inter-rater agreement, SSIS

## Abstract

Objectives: This study explores the patterns of agreement and discrepancy among informants (teachers, parents, and students) in the domains of the Social Emotional Skills Scale Assessment System—Social Skills Scales (SESAS-SS), which is a translation of the Social Skills Improvement System—Rating Scales (SSIS-RS) for use in Spain. Methods: The sample is composed of students, 88 teachers, and 98 parents from Spanish secondary schools. Inter-rater agreements have been assessed, calculating the Pearson correlation coefficients among pairs of raters, effect size indices, and intraclass correlation coefficients at the subscale and total scale level. Results: The convergent validity coefficients were stronger than the divergent ones, with the highest level of agreement between teachers and parents in social skills, particularly for total social skills, engagement, empathy, and communication. The patterns of discrepancies confirmed weaker agreements between teachers and parents in self-control and between parents and students in empathy. Significant differences were also found in students’ estimates depending on gender. Conclusions: The SESAS-SS provides support for previous studies on inter-rater agreements for SS, extending the focus on the degree of agreement in the estimate of dyads of raters when considering the students’ gender.

## 1. Introduction

Social skills (SS) involve learned behaviors that promote positive interactions while simultaneously discouraging negative ones when applied to appropriate social situations [1]. They constitute a specific class of behavior an individual follows to successfully perform a social task, as opposed to social competence, which is an evaluative term based on the consideration (given certain criteria) that an individual has performed the social task adequately [2]. This means that SS include interpersonal behaviors that either facilitate or impede interactions with others within a given social environment [3].

Theoretically, research on children’s social and emotional skills has been substantially influenced by Bandura’s social learning theory [4] and currently is influenced by more applied models of social–emotional learning such as that advanced in the CASEL competency framework [5]. These approaches to characterizing social–emotional behavior emphasize clusters of social skills that are largely observable behaviors expected to correlate positively with prosocial outcomes and academic performance, while correlating negatively with emotional behaviors such as internalizing and externalizing [6].

Social skills’ developmental needs may vary within and across family and school contexts, mainly because these contexts are different and include contingencies. Furthermore, paraphrasing De Los Reyes et al. [7], contingencies central to addressing students’ needs might manifest cross-contextually or in context-specific ways: interactions with peers at school might contain aversive or hostile factors (e.g., teasing) that students do not encounter at home. Because contingencies inform the planning of the social and emotional education that students receive, these authors point out assessments used to guide decision-making, which must leverage information sources that, collectively, harbor the ability to ably “track” subjects’ needs within and across contexts. The needs of two students are not manifested exactly the same, even when their needs emerge from the same domain. In this sense, the multi-informant approach to assessment commonly provides multiple discrepant estimates of students’ educational needs.

Multiple informant agreement has become a salient part of these theoretical and applied approaches because they each recognize the importance of the social environment on the behavior children develop and exhibit. That is, successful development of social skills involves children learning to discriminate what are appropriate and effective behaviors in different settings. Two of the most important settings for children are home and school, hence the high interest in using parents and teachers, along with children themselves, in the assessment of social skills across multiple settings [6].

One of the main instruments in the assessment of SS, the SSIS-RS [1], has been culturally adapted in Spain for research purposes referred to as the Social Emotional Skills Scale Assessment System—Social Skills Scales (SESAS-SS). This Spanish version of the SSIS-RS is a content-valid measure of social–emotional skills for adolescents [8]. SS are context-specific [9,10], and the inclusion of numerous informants—each one with their own values and focus on particular aspects of SS—allows exploring the substantial variability among informants’ perceptions [11]. It therefore seems expedient to study the Spanish cultural context to discover whether similar levels of (dis)agreement are found among different types of informants (similar and consistent ratings for certain subscales compared to others, and absolute (dis)agreement).

This kind of multi-informant diagnosis would have a more positive impact in educational contexts because although students’ self-reports have provided sufficient proof of diagnostic validity, in line with Brinkworth et al. [12], the opinion that students and teachers have of their own relationships is an important environmental aspect that can promote social and emotional learning (SEL). The results from varying ratings can influence the comprehensiveness of the assessment of social skills and social–emotional learning and the planning of intervention for its improvement. We can use this measure now to evaluate the effects of programs in schools. The 7.1. recommendation from the analytical report, “A formative, inclusive, whole-school approach to the assessment of social and emotional education in the EU”, notes there is a need to clearly identify the key social and emotional education (SEE) competencies in order to plan and assess learning accordingly [13]. The inclusion of the “Personal, Social and Learning to Learn” as a Key Competence for lifelong learning [14] has begun an ongoing process with regard to the effective integration and implementation of SEE in curricula across the Member States. Co-funded by the Erasmus + Programme of the European Union, the Promoting Mental Health at Schools (PROMEHS) Project was carried out to provide a systematic framework for the development and implementation of an evidence-based universal mental health curriculum in schools (to enhance the social and emotional well-being of students, improving their attitudes towards the self, others, and learning, as well as reducing conduct problems and aggression, emotional distress, and anxiety) and to deliver high-quality training for school staff. The Social Skills Improvement System Social Emotional Learning Edition (SSIS SEL RF) and SELA [15] was used in this project, providing scores for the five CASEL domains (self-awareness, self-management, social awareness, relationship skills, and responsible decision making) as well as three academic learning domains (motivation, reading, and mathematics). Thus, teachers were able to evaluate students’ social and emotional competencies using a combination of these domains to identify the students’ strengths, as well as areas that require further development and instruction [13].

Many educators and psychologists are interested in both measuring and improving children’s SEL skills, but without sound multi-informant assessments with known and sound psychometric evidence, many fundamental questions about children’s SEL skills will go unanswered. Sundry prior studies on patterns of agreement among teachers, parents, and students in the domain of social–emotional skills and their ratings of behavioral and emotional problems have found weak to moderate levels of agreement that depend on the informant’s role in a young person’s life, the type of behavior observed—externalizing or internalizing—or their more or less problematic nature. Although this study is not designed to directly address the debate in the literature concerning the meaning of the discrepancies among multiple informants or the several methodological approaches to analyze the inter-rater discrepancies, we agree with [16] that there is no universal gold standard in handling inter-rater discrepancies [9,11,16,17].

Discrepancies may inform the characterization of students’ psychosocial functioning and facilitate the identification of more precise and effective evidence-based services [18]. Discrepancies in the ratings tend to be attributed to the different environments in which a young person’s behavior is observed. Indeed, certain SS could be highly context-specific [19], as schools give rise to numerous scenarios that require students to use their skills to negotiate differences with others, which is not necessary in a more familiar and probably more relaxed setting, such as their home. In fact, the SS required to successfully function at home may not be the same as those called for at school. In turn, parents’ beliefs and expectations about their children may be different from those held by teachers [20] and have an influence on patterns of social interaction, especially when other variables are considered, such as household income, whether the student is normo-typical [21], their performance, or the way in which this may influence the perception—more positive or negative—that a teacher has of their students [12]. Even when events are nominally identical, parents and teachers may view the same behavior and interpret events differently, as raters attend to the conditions that elicit behaviors [22]. In addition, cultural values may also modulate the importance that informants give to different SS, as reported by Mudarra and García-Salguero when finding that in terms of academic success, both Spanish and US teachers assigned considerable importance to cooperation [6,23], yet they differed in their rating of self-control and assertion. Elliott et al. [24] found that parents’ observations of their children’s social–emotional skills based on social interactions within a family are potentially rich, but parents typically have not been included in universal SEL screenings conducted by schools. It appears that there are variables and meanings that are not shared among dissimilar informants, whereby, if feasible, it would be most expedient to use different sources of information in the diagnosis of SS.

From a methodological viewpoint, this study seeks to overcome one of the limitations involving the different metrics used by raters (frequency vs. true/false), identified by Gresham et al. [25], as well reduce acquiescent responding, since in the Spanish version of the SSIS, all the SS forms—students, teachers, and parents—share the same scale (frequency). To allow replicating [2,9,25], an effect size index and methods from the cross-informant agreement research literature were included. Furthermore, the inclusion of intraclass correlation coefficients—ICC—for assessing not only the consistency between ratings but also the extent to which raters’ individual scores actually match (absolute agreement) raters for one of the considerations was not addressed by Gresham et al. [9] regarding item-level agreements between raters.

The purpose of the study was therefore to systematically explore patterns of agreement and discrepancy among teachers, parents, and students in domains of SS Spanish forms. This investigation used a Spanish adaptation of the revised Social Skills Rating System (SSRS) [25], now known as the Social Skills Improvement System—Rating Scales (SSIS-RS) [1]. We hypothesized that pairs of informants (teacher–parent, teacher–student, and parent–student) would record greater than chance levels of agreements, as indexed by significant inter-rater Pearson r correlations, especially higher teacher–parent correlations than teacher–student and parent–student pairs across social skills domains [25,26,27].

## 2. Materials and Methods

### 2.1. Participants

The sample consisted of 1125 secondary students selected from the adaptation cohort of the SESAS [8], with the inclusion criterion being that they had other complete ratings of their social behaviors from informants (teachers, parents) on respective SESAS rating forms. Two sub-samples were formed with 88 teachers and 98 parents, respectively.

The sample was obtained from the adaptation cohort involving an intentional stratified selection by educational area (North, 31%; South, 30%; East, 13%; and West, 26%). The reference population of students enrolled in secondary education in the Autonomous Community of Madrid (*n* = 409,479) required a representative sample of 1064 students (95% confidence level with a 5% error, p = q = 0.50). The final size of the adaptation sample (revision version of SSIS-RS in the Spanish cultural context) was *n* = 1668, as only 12 of the 22 schools accepted the written invitation to take part in the adaptation study.

Table 1 shows the demographic information for the participants. The student sample was evenly balanced as regards gender (males *n* = 563, and the binomial test indicated that the proportion of males and females, 0.50, was more balanced than expected, *p* = 1).

To test whether males and females differed in terms of SS, a series of Mann–Whitney U Tests following Field [28] were performed (see Supplementary Material File).

Most students were White (*X*^2^ (2) = 2,039,353, *p* = 0.000), with an average age of 14.2 (SD = 1.5, range = 11–22) with highest educational level corresponding to middle school (*X*^2^ (2) = 464,368, *p* < 0.001). The proportion of state schools, 0.42, was lower than the expected 0.50, *p* < 0.001. Most of the teachers were female (*X*^2^ (1) = 5.500, *p* = 0.019) with fewer than five years’ experience (*X*^2^ (2) = 38,979, *p* = < 0.001) and a broad range of knowledge areas. Finally, mothers’ reports prevailed (their proportion of 0.72 was higher than the expected 0.50, *p* < 0.001).

### 2.2. Instrumentation

The Social Emotional Skills Scale Assessment System—Social Skills Scales (SESAS-SS) [8] is the cross-cultural adaptation of the Social Skills Improvement System—Rating Scales (SSIS-RS) [1] for assessing social and emotional behaviors (SS) in the Spanish cultural context—a slightly revised version. Like the SSIS-RS, the SESAS is a multi-informant series of rating scales that involves teachers, parents, and students. All the forms include common SS in their domains [9]. This research considered solely the data gathered on the frequency of items on the SESAS-SS scale/subscales. Specifically, the Problem Behavior scales from the Parent, Student, and Teacher versions of the RS were excluded along with the Academic Competence Scale on the Teacher version. The resulting SESAS-SS consisted of seven subscales, measuring SS in the domains of Communication, Cooperation, Assertion, Responsibility, Empathy, Engagement, and Self-Control, which are combined in an overall scale that examined these skills from the perspectives of the students (student form, SESAS-SS-S), the teachers (SESAS-SS-T), and parents/guardians (SESAS-SS-P). The item rating anchors for the student, teacher, and parents forms all applied a 4-point Likert scale—0 (never), 1 (seldom), 2 (often), and 3 (almost always)—to rate the frequency with which students use each social skill. This use of a frequency scale for the Student version is a change from its 4-point Not True to Very True. The scores for the composite scale (SS) are the aggregate of all the subscales (scores for subscales are raw scores).

### 2.3. Procedure

Participants gave their written informed consent and completed the questionnaires either online through Google Forms links on PCs or iPads (Student forms), or with a pencil and paper test (Teacher and Parent forms). The SESAS takes about 15 to 20 min to complete in a single session. Participants were identified through a common code for students, teachers, and parents to ensure their anonymity. Missing responses from teachers and parent forms were handled via the guidelines recommended by SSIS-RS developers, multiplying the number of missing items by the adjustment factor according to the maximum number of missing items allowed [1]. Students, teachers, and parents were not provided with any systematic incentive to complete the online versions of SESAS.

### 2.4. Data-Analytical Strategy

Consistent with the analytical strategy following the original instrument [9,25], we first calculated correlations between pairs of raters for analyzing the patterns of agreement (teacher–parent, teacher–student, and parent–student). Although the scores of these three forms (teacher, parent, and student) met the assumption of multivariate normality in the SSIS SEL RF, the most recent proposal made by Gresham et al. [26,27], there is insufficient theoretical or empirical evidence to affirm that the scales and subscales of the SSIS-RS, and therefore of the SESAS, follow a normal distribution [1]. The Pearson correlations between sets of common items provided by different raters were calculated for comparing with these prior studies [9,16,26,27].

The pattern of the findings was practically the same when Spearman’s nonparametric correlations were used. Bivariate correlations were calculated to examine the convergent and divergent relationships for evidence of inter-rater reliability. Convergent correlations were those between the ratings for the same subscale (e.g., teacher communication–parent communication, parent empathy–student empathy, etc.). Divergent correlations were those between raters for different subscales (e.g., teacher communication–student engagement, parent cooperation–teacher empathy, etc.). Following Campbell and Fiske [29], the measures of theoretically similar constructs should be highly intercorrelated, while those of theoretically different constructs should not correlate highly with each other. Convergent correlations should be greater than divergent ones (although different measures of an SS scale could be interrelated due to the shared trait variance). Thus, the level of agreement was analyzed in all the subscales and on the total SS scale.

Fisher’s z transformation was used to test the significance of the differences between the correlation coefficients. As it was not possible to directly calculate the mean of the Pearson coefficients (as the distances between correlations need not be the same) for comparing the correlations, they were converted into a common metric, transforming them by means of Fisher’s Zr (z’ = 0.5[ln(1 + r) − ln(1 − r)]). The means were calculated using these new transformed correlations involving the following formula in Excel: FISHERINV(z) = (EXP(2 * z) − 1)/((EXP(2 * z) + 1)). Finally, the p-values were set at 0.05, and data were analyzed using the SPSS statistical package version 27, G*Power version 3.1. and Excel.

The second index for examining inter-rater agreement was effect size. The standardized mean difference effect size is the discrepancy between raters—the extent the dyad agreed—on the total SS scales and subscales in standard deviation units to allow comparability with prior studies [9,26]. According to these scholars, effect sizes approaching less than 0.20 indicated high agreement between raters, whereas effect sizes reaching 0.80 indicated high discrepancy, and the effect’s sign or directionality is interpreted according to the following: positive values are assigned to effect sizes to indicate that the adult (parent or teacher) provided less favorable ratings of SS than the student. For the teacher–parent dyad, and following Gresham et al. [9], positive values were used to indicate that teachers provided less favorable ratings on SS than parents.

To complement the Pearson correlation analyses by reflecting both degrees of correlation and the level of agreement between measurements, calculations also were made of the intraclass correlation coefficients (ICC) between teachers and parents, teachers and students, and parents and students, at both subscale and total scale level. ICC estimates and their 95% confidence intervals were calculated using the SPSS statistical package version 27. Following the recommendations made by McGraw and Wong [30], the type of ICC and the definition of the relationship considered involved a two-way random-effects model based (average) on absolute agreement. The interpretation of the ICC applied the criteria indicated by Cicchetti (1994) (ICC < 0.40, poor agreement; 0.40 < ICC < 0.59, fair agreement; 0.60 < ICC < 0.74, good agreement; and ICC > 0.75, excellent agreement)

## 3. Results

The translation of an established social behavior assessment and examination of the validity of scores from the assessment generates a substantial amount of evidence in pursuit of addressing terminal questions concerning inter-rater or cross-informant ratings. Our questions and related analysis in this section focus on the reliability of scores on the new assessment using three types of estimates: internal consistency, inter-rater correlations, and ICCs. Of these, the internal consistency and inter-rater correlations are valuable for their comparison to previous findings with the original English version of the SSRS. The results involving the ICCs are the unique and featured findings.

### 3.1. Reliability

The SESAS-SS recorded good reliability in this study, with all αs > 0.7 [31] (see Table 2). Internal consistency estimates for total SS scores were 0.95, 0.97, and 0.91 for teachers, parents, and students, respectively (with median coefficients of 0.89, 0.85, and 0.70). In turn, as regards the validation sample for the SESAS-SS, the internal consistency estimates were 0.97, 0.95, and 0.90 for the total SS scores (with median coefficients of 0.89, 0.85, and 0.70) for teachers, parents, and students, respectively. The SS scale tends to record values of α of more than 0.90, as with the SSIS-RS [1,9,32,33]. The most reliable subscales in the forms of teachers, parents, and students (see Table 2) were empathy, engagement, and self-control, respectively. By contrast, again in each one of the forms, the subscales with the lowest αs were communication (teachers) and assertion (parents, students). These estimates of reliability reflect those recorded in the validation sample and are consistent with SSIS-SR alpha coefficients [1,9,32,33]. As with the SSIS-RS [9], validity evidence for the SESAS-SS has been provided by factorial analyses [6], which ratify its structure, and correlational studies with the Behavior Assessment System for Children [34,35], which records lower to moderate correlations, particularly with clinical subscales.

### 3.2. Agreement between Pairs of Raters

The results of the concordance of Teacher–Parent (Table 3), Parent–Student (Table 4), and Teacher–Student (Table 7) are discussed below. These results are followed by standardized mean difference effect sizes for different between-raters’ scores on Total Social Skills Scale (Table 5) inter-rater agreement of Social Skills Scales for SESAS-SS scores (Table 6) and a comparison of Social Skills with the stronger and weakest agreement across raters and studies (Table 8).

#### 3.2.1. Teacher–Parent Agreement

The convergent correlations between the estimates of teachers and parents for the sub(scale) of SS—see the diagonal of the correlations matrix in Table 3—ranged from a minimum of −0.07 (self-control) to a maximum of 0.525 (total SS) (See Supplementary Material, Table S1. Correlations Teacher-Parent (subscales and total, confidence interval included)). Four subscales record non-significant correlations: empathy, cooperation, assertion, and self-control. Nevertheless, the results reveal significant agreements between teachers and parents in the estimates on total SS, engagement, responsibility, and communication. Furthermore, the mean of convergent correlations was slightly higher than that for divergent ones, as the average for Fisher’s Z-transformation for convergent correlations was 0.414, with a mean r of 0.39 (95% CI [−0.12, 0.94]), while the same average for the divergent ones was 0.35 with a mean r of 0.35 (95% CI [−0.16, 0.9]). Except for certain exceptions—such as the correlations parent cooperation/teacher responsibility, parent empathy/teacher communication, and parent social skills/teacher communication—the convergent correlations tend to be stronger than the divergent ones, although they record moderate convergent validity indicators for the SESAS-SS.

The standardized mean difference effect size for the parent–teacher dyad (see Table 5) for the overall level of SS was 0.00 and 0.01 in its estimates regarding male and female students, respectively. As regards the sign, and in both cases, teachers provided slightly less favorable ratings than parents. Following the guidelines provided by Cohen [36] and Sawilowsky [37], the effect size for students is very small in both genders, indicating high agreement between teachers and parents in their ratings of SS.

In turn, regarding the ICC between teachers and parents (see Table 7), close agreements were found for several subscales: engagement (0.69) and empathy (0.60); reasonable agreements were found for communication (0.59) and SS (0.49); while no significant agreements were found between teachers and parents in all the other (sub)scales.

#### 3.2.2. Parent–Student Agreement

All the convergent validity indices on the diagonal in Table 4 were significant, ranging from a minimum of 0.20 (empathy) to a maximum of 0.43 (total SS), which means they reflect weak to moderate consistencies in the ratings of parents and students on the (sub)scales. Once again, the average of the convergent correlations was slightly above the mean of divergent correlations, as the average for Fisher’s Z-transformation was 0.34 with a mean r of 0.33 (95% CI [0.14, 0.54]), while the same average for the divergent ones was 0.25 with a mean r of 0.24 (95% CI [0.05, 0.45]) (See Supplementary Material, Table S2. Correlations Parent-Student -subscales and total, confidence interval included)). Therefore, when parents and students rate the same (sub)scales of SS, they express a higher level of agreement than when they rate different (sub)scales, recording convergent validity indicators for the SESAS-SS that tend to be moderate.

The standardized mean difference effect size for the parent–student dyad (see Table 5) on the overall level of SS in its estimates regarding male and female students was −0.22 and 0.16, respectively. As regards the sign, in this case there are indeed differences depending on the students’ gender, whereby the ratings of male students are less favorable than those of their parents, while the opposite is true in the case of their female peers. Furthermore, the effect is small yet not insignificant in the case of male students, and very small for females, which means there is close agreement in the estimates between parents and female students regarding their SS, with close agreement also, only slightly less so, between parents and male students.

ICCs were statistically significant (*p* < 0.05) for all the (sub)scales. The highest ICCs (see Table 6) were found for total SS (0.60), indicating close agreement. Reasonable agreement was found for engagement (0.53), assertion (0.51), communication (0.45), and self-control (0.44). The lowest values—weak agreement—were found for empathy (0.33), cooperation (0.35), and responsibility (0.38).

#### 3.2.3. Teacher–Student Agreement

The only significant convergent validity index (see diagonal of the matrix of correlations, Table 7) corresponded to the cooperation subscale, with correlations ranging from a minimum of −0.015 (empathy) to a maximum of 0.386 (cooperation), indicating weak convergent validity between teachers and students in most of the (sub)scales (See Supplementary Material, Table S3. Correlations Teacher -Students (subscales and total, confidence interval included)). Even with very weak correlations, the means for convergent correlations continued to be slightly higher than for divergent ones, as the average Fisher’s t-transformations in the convergent cases was 0.10, with a mean r of 0.1 (95% CI [−0.11, 0.31]), while the average Fisher’s t-transformation was 0.02, with a mean r of 0.02 (95% CI [−0.19, 0.23]). This difference between convergent and divergent correlations continues to reflect, in general, a higher level of agreement among teachers and students when rating the same (sub)scales of SS. When rating different constructs, however, the SESAS-SS indicators of convergent validity tend to be weak.

The standardized mean difference effect size for the teacher–student dyad (see Table 5) about the overall level of SS was −0.22 and 0.18 in its estimates regarding male and female students, respectively. This upholds the gender-related differences in the effect size for the teacher–student dyad. Male students’ ratings of their SS are less favorable than those of their teachers, yet the opposite is true in the case of female students. Once again, the effect size is small in the case of male students, and somewhat lower in the case of their female peers, which means there is close agreement in the estimates between teachers and female students, while that agreement, although still high, is slightly lower between teachers and male students.

Only one ICC was statistically significant (*p* < 0.05), indicating reasonable agreement between teachers and students for the cooperation scale.

## 4. Discussion

### Key Findings

The following points highlight the key results of the study. The reliability of the SESAS-SS is good with all αs > 0.7. In accordance with the patterns of agreement and discrepancy among teachers, parents, and students, it may be concluded that agreements are greater than disagreements with closer teacher–parent correlations (particularly on total SS, engagement, responsibility, and communication) than teacher–student (the dyad with biggest disagreements) and parent–student ones. From a gender perspective, females in this study were consistently rated significantly higher on most SS, and their parents provided a more positive rating of their SS than the parents of male students.

These results are consistent with prior studies [9,26,27,38], as overall, teachers, parents, and students do not perceive their level of SS differently. Indeed, a comparison of the correlations between this study and the prior ones cited—using the median test as indicated by Rosenthal and Rosnow [39]—confirms that the results are statistically the same (*Mdn p* = 0.721). Moreover, this study rejects the independence of correlations between rater dyads (*Mdn p* = 0.223).

These findings support both our first and second hypotheses, as pairs of different types of informants (teacher–parent, teacher–student, and parent–student) recorded greater than chance levels of agreement, with closer teacher–parent correlations than teacher–student and parent–student ones. Specifically, past researchers have found parent–teacher correlations of 0.30, 0.33, and 0.38; parent–student correlations of 0.21, 0.29, and 0.25; and teacher–student correlations of 0.21, 0.23, and 0.21 [9,26,38,40]. This study has found a parent–teacher correlation of 0.39; a parent–student correlation of 0.33; and a teacher–student correlation of 0.01. Again, following Gresham et al. [9], a comparison of these correlations, using a dependent correlations test, showed that they were not statistically different in this study either (parent–teacher, *p* = 0.34; parent–student, *p* = 0.50; teacher–student, *p* = 0.37). These results are consistent with the prior studies cited, although the agreement correlation between teacher–student in our study, while significant, is still very small.

Considering the size of the agreements between different informants in the various (sub)scales, the results of this research with the SESAS-SS provide evidence to support that convergent validity coefficients are consistently stronger than divergent ones, yet as in prior studies [9,26], the indicators of convergent validity were weak to moderate. The reason for this moderate convergent validity might be that the different constructs reflected in the subscales share variance with a broader and more complex construct, namely, SS. The differences among pairs of raters in the (sub)scales—analyzed through effect size—were small or very small, reflecting high levels of agreement between informants. In particular, the highest levels of agreement in SS were found between parents and teachers (as in Gresham et al. [9], with the less favorable ratings by teachers mirroring other studies [17]).

An interesting result involves the gender-based differences found in the ratings when one of the informants was the actual student; that is, in the P-S and T-S dyads. No significant gender-based differences were found in the ratings of teachers and parents, although they were found in students’ estimates in certain (sub)scales. Accordingly, the agreements between parents and female students and between teachers and female students were high and significant. They were also high—albeit slightly less so—in both cases when the students were male, as they rate their SS less favorably. Taking into account, furthermore, that female students recorded values that were slightly but significantly higher than their male peers in SS, communication, and cooperation, these results may be taken as proof of the validity of SESAS-SS’s scores, as the students’ scores in their self-reports match the ratings of teachers and parents (although a student’s gender did not have a significant impact on the individual estimates of teachers and parents).

These findings also are in line with the results of the inter-rater absolute agreement (ICC). This index also provided information that complemented the Pearson correlation, showing good and reasonable agreements between teachers and parents, especially in the subscales of engagement, empathy, communication, and SS. Nevertheless, the Pearson correlation between the estimates of teachers and parents in empathy was not significant. Given that prior studies [9] have found this relationship to be small but not insignificant, the ICC in this case reveals the need to continue exploring the agreements between teachers and parents in the empathy subscale. Regarding the agreements between parents and students, the ICC—confirming the significance of the Pearson correlations—revealed good and reasonable agreements in SS, engagement, assertion, communication, and self-control. Furthermore, this index of total agreement between teachers and students, which is significant solely in the cooperation subscale, ratified the only significant index of convergent validity found between teachers and students in that same subscale. Another result of note involved the weak agreements between parents and students in empathy, cooperation, and responsibility, and especially the minimum convergent validity coefficient between these informants in the empathy subscale.

As expected, these results are consistent with the study by Gresham et al. [9] that addressed the rater combination and type of subscale with different impacts on cross-informant agreement estimates, although some agreements/disagreements recorded different sizes and/or different (sub)scales. Stronger convergent validity estimates were found for teacher–parent ratings, particularly in the subscale of engagement, again with high and significant agreements in responsibility and SS, and weaker ones in self-control (see Table 3 and Table 8). Again, in keeping with Gresham et al. [9], significant and similar agreement values were recorded in responsibility (around 0.3) between parents and students, and greater discrepancies in empathy (see Table 4 and Table 8) Precisely in a study with the SSIS SEL Rating Form, Elliott and Alvarez-Gonzalez [2] highlight the value of multi-rater assessments because students and adults are likely to disagree with regard to self-awareness and social awareness skills.

This means that the same occurs regarding emotional and behavioral problems; the reports of parents and their children do not appear to be interchangeable [41]. Although there was surprising agreement between parents and students in the subscale of cooperation, recording similar figures to those reported by Gresham et al. [9], the ICC in this study recorded a weaker agreement in this subscale. This may be because adolescents tend to think they are engaging in SS more frequently than their parents [42], or it might be reflecting differences in the perception of this construct—expectations—by teachers and parents. As Beebe-Frankenberger et al. [22] have reported, teachers estimate behavioral problems almost entirely in the cooperation domain, rating skills that display assertion and self-control as less important, while parents value behaviors at home in the domains of self-control, responsibility, and assertion, rather than in cooperation.

The weakest estimates in our study (as in Rupp et al. [43]) were found for the teacher–student dyad, not between parents and students, as was the case in Gresham et al. [9,26] and Gresham et al. [26]. It is possible, as West et al. contend [40], that many teachers tend to assess students holistically rather than draw distinctions between different constructs. This study found that the highest scores in SS tend to be awarded by parents, students, and teachers, in this order. A comparison between the scores given by students and adults (whether parents or teachers) shows that female students assign higher scores than adults. By contrast, male students’ estimates are lower than those recorded by adults. This latter result, also reported by Gresham et al. [44], was related to the low frequency at which any rater estimated, for a given adolescent, more than one SS acquisition deficit (possibly because the SSIS-RS does not have sufficient “scope” to reliably detect base rates of SS acquisition deficits across ages and raters). Nevertheless, more recently, McMahon and Solomon have reported that adolescents both estimated that SS were less important and engaged in SS more often than their parents reported [42].

In our opinion, this diversity of results in the SESAS-SS is not due to differences in the scale of metrics between the various forms, as all the SS forms—students, teachers, and parents—share the same one (frequency). Instead, the explanation might be related to differences in the perception of SS between informants and the variables moderating their ratings, as more or less explicit beliefs, values, or aspects involving the cultural context. Rupp et al. [43] likewise found higher levels of consistency in ratings by parents and students than by teachers and students for perceived bullying behavior, indicating that the differences across informant scores pointed to students’ scores being perceived completely differently and noted the importance of context when considering ratings by different types of informants. As these scholars have stated, “The value of understanding the informant’s perceptions about the student’s behavior could provide answers as to why the student’s scores vary in different ways [43] (p. 463)”.

The closer coincidence in the P-S estimates than in the T-S ones suggests there may be differences between teachers and parents/students in the interpretation of the students’ internal dispositions involved in SS and the way in which these are transferred to different contexts (with this transfer being more readily observed by parents than by teachers). The highest level of agreement between teachers and parents implies not only a more efficient social and emotional assessment but a common starting point to collaborate in the implementation of proactive practices to improve these skills at school and at home. The highest ratings among teachers and students on the cooperation subscale (a relevant construct in a school setting) might mean that the more significant agreements between teachers and students occur precisely in those behaviors that are more readily observed. Moreover, the significance attributed to these directly observable behaviors, and therefore their estimation in terms of SS, might also vary depending on the cultural contexts each informant may be exposed to. Carneiro et al. [45] refer to the influence of contextual variables on the disagreement among informants when studying the reports on emotional and behavioral problems.

In this study, more discrepancies were found among teachers and parents in the self-control domain, parent and students in empathy, and, in general, more small discrepancies between teachers and students than between parents and students [9]. Disagreement among informants does not necessarily indicate unreliability in one or both informants; conversely, this may indicate that different informants contribute with complementary information [46]. Students differentiate more among emotional inclusion, social inclusion, and academic self-concept, whereas mothers, fathers, and teachers differ less between these dimensions of students’ inclusion at school; thus, it matters who reports students’ emotional inclusion, social inclusion, and academic self-concept [47]. There are certainly behaviors that can be perceived as very socially effective in the family and not so much at school, and vice versa. Furthermore, the informants may vary in their backgrounds, characteristics, and as pointed out by De Los Reyes et al. [7], expertise for observing students within specific contexts. In the case of youths, observations of their own behavior pass through the contexts observed by parents and teachers, and they might also include contexts unique to themselves (e.g., peer interactions that neither parent nor teacher observes) [48]. Looking at trajectories of informant discrepancies and associations with personality types, Mastrotheodoros et al. [49] have shown that maternal and paternal personality types were associated with parent–adolescent discrepancies differently (father–adolescent discrepancies were predicted by both adolescent and paternal personality). In addition, in relation to emotional and behavioral problems, perhaps the parenting stress may confound reports [50].

Accordingly, the informants’ discrepancies partly indicate the differences in their experience or knowledge about the context or contexts that surround young people’s needs, but they can also reflect other characteristics of the different informants. Hence, the high interest in counting on parents and teachers, along with children themselves in the assessment of social skills and across multiple settings [26]. It is essential to preserve the contributions of different informants, even if their reports are not highly correlated [51], as through the interpretation of multi-informant data, we will be able to achieve the optimal educational interventions.

Thus, social and emotional competencies are better assessed using multiple sources and various modes of assessment [52]. Multiple sources including self-, teacher, peer, and (where appropriate) parent assessment are particularly useful in SEE, where the behavior observed is often evaluator- and context-specific [53]; multi-informant rating systems are thus the most feasible tool for use in the classroom.

## 5. Limitations and Future Directions

Our results clearly support prior studies with the SSIS-RS [9], yet this study has several potential limitations, as it relied on a nonprobability sample of participants, although a stratified intentional sampling was made of the Autonomous Community of Madrid’s Educational Area. Along these lines, despite the sample’s suitable size, its share of state schools was lower than expected, thereby hindering the generalization of the results.

Despite these limitations, these results suggest several theoretical and practical implications. According to the OECD [54], teachers and parents may not know whether their efforts at developing skills such as sociability are paying off, and what they could do better, among other reasons, because they perceive that social and emotional skills are hard to improve if they can in fact be taught and honed. Elliott and Álvarez-González [2] have calculated base rates of social–emotional learning (SEL) skill deficits and strengths to create a developmental description for benchmarking and contextualizing accurate decisions about which skills are likely to need more attention for improving their rate relative to their naturally occurring rate. As Solberg et al. report [55], future SEL programs will need to focus on developmental approaches that understand individuals’ existing strengths and qualities.

Certain considerations of this study could be addressed in future research. To ensure that these assessments meet the needs of their users, it is essential to explore ways of using the information produced by the high-quality assessment of social–emotional skills [56]. The OECD has specified that “more efforts need to be made to improve measurement instruments so that they are robust to inter-cultural and linguistic diversities and response styles” [54] (p. 2).

This study is a first attempt to address the patterns of agreement and discrepancy among teachers, parents, and students in the domains of SS in SESAS-SS, a Spanish revised version of the Social Skills Rating System in the Spanish cultural context [25], but we feel that further research may be helpful. Our findings here provide proof of score validity for the use of the SESAS-SS [8] in the Spanish cultural context. This study confirms a higher level of agreement in the estimates on SS among teachers and parents than in other dyads (parents–students and teachers–students). Nonetheless, as noted by Achenbach [51], the size of these agreements—from weak to moderate—is probably reflecting part of the variance that is not shared across the different types of informants. Therein lies the importance of discovering the mechanisms or factors creating informant discrepancies [9] and analyzing in depth the students’ social experiences and their reaction to them [20].

It would be useful to extend the current findings by examining the level of agreement between the same kind of raters (e.g., teacher–teacher, parent–mother) to discover when this agreement is higher than that between dissimilar informants, which might shed some light on the diversity of the informants’ roles and contexts and their impact on the estimation of SS. As reported by Major et al., “ratings may be perfectly reliable and valid, although different” [57] (p. 4), and discrepancy may be as instructive as agreement [51]. To appropriately address the matter of discrepancy between different types of raters, for example, future research can record a series of videos of the same children in different settings over the course of 3 or 4 weeks, then have both the parent/mother and teacher and a third-party observer watch the video and then complete the rating forms. Another methodological approach is the analysis of the agreements at the item level through research designs that include qualitative analyses, which may also shed light on the way different types of informants interpret the SS observed as well as the influence that personal and contextual variables have on said interpretations. Highlights among these variables are the impact of socio-demographic drivers such as household income, which Heyman et al. [17] have shown to be significant through the SSIS’s five subscales [1], family characteristics [58], and the context—home vs. school—in which this behavior is observed [18].

Finally, if, as the present study suggests, there are significant differences in SS estimates due to the students’ gender, it would be pertinent to discover whether male students have fewer expectations regarding their SS, and therefore tend to assign lower scores than their female peers, prompting a reference bias. Although Elliott and Álvarez-González [2] did not find any significant differences between a sample of US males and females regarding SEL acquisition deficits, performance deficits, or strengths, females in this study were consistently rated significantly higher on most SS, and their parents provided a more positive rating of their SS than the parents of male students.

There is a need for research that explores the existence, or otherwise, of gender-based biases in these kinds of diagnostic instruments and the interactions between the students’ gender and that of the teachers and parents rating their SS.

## 6. Conclusions

Following and extending a prior research stream on cross-informant agreements for SS whose results show that such agreements tend to be weak to moderate, a Spanish translation of the SSIS-Rating Scales, called the SESAS-SS, has closely replicated previous findings with parents, teachers, and students in the Spanish cultural context of Madrid. Spain, as a member of European Union, intends to make mental health and well-being a global development priority in schools as well. How social and emotional competence is manifested, how it should be assessed, and to what extent the assessment reflects such cultural variations is a key issue [5]. Significant cross-cultural differences were found in social and emotional education (SEE) provisions as well as in teachers’ beliefs about the purpose of SEE [54]. As Europe becomes more socially and culturally diverse, the need for culturally responsive assessment that makes use of flexible and multiple forms of assessment becomes more salient [13]; policymakers and practitioners need to ensure that the SEE assessment tools being used are not biased against particular groups of students as a result of socio-cultural differences, both in terms of how assessment is carried out and whether the underlying construction of SEE reflects the relevant cultural variations [59,60].

The findings contribute to a growing body of evidence on SS rating scales, replicating previous research [9,24,26,27] and providing support for their cross-cultural use. We trust that this research will stimulate further investigation of this important area not so much in the search for perfect levels of agreement between multi-informants in their estimates of SS but instead in a greater understanding of the factors with a bearing on their estimates, and in the education needs and decisions arising from this understanding. This investigation replicates studies elsewhere in English with different cultures, and it seems that the underlying constructs of social–emotional functioning seemed to hold up across these cultures, which seems interesting. This study advances work in Spain and Spanish cultures and contributes to international comparisons.

## Figures and Tables

**Table 1:** Demographic characteristics.

Characteristics	*n*	%
Student form		
Gender		
Male	563	50
Female	562	50
School Type		
State	471	41.9
Private	654	58.1
Highest educational level		
Middle school	587	52.2
High school	500	44.4
Vocational and technical school	38	3.4
Ethnicity ^a^		
White	1081	97.1
Latinx	31	2.8
Asian	1	0.1
Teacher form		
Gender ^b^		
Male	33	37.5
Female	55	62.5
Experience (Years) ^b^		
<5	35	74.5
5–10	11	23.4
>10	1	2.1
Knowledge area ^c^		
Biology	1	2.13
English	11	23.4
History	13	27.7
Maths	14	29.8
Philology	4	8.5
Language and Literature	3	6.4
Art	1	2.13
Parent form		
Gender ^d^		
Male	22	28.2
Female	56	71.8

Notes: Students, *n* = 1125. Teachers, *n* = 88. Parents, *n* = 98. ^a^ *n* = 1113; ^b^ *n* = 57; ^c^ *n* = 47; ^d^ *n* = 78.

**Table 2:** Domains measured by the SESAS-SS along with sample items and reliability estimates.

						α			α (Gresham et al., 2010) [9]		
Scale/Subscale (Number of Items)	Example Items	Example Items (Spanish Adaptation)	No, Items			Teacher	Parent	Student	Teacher	Parent	Student
Social Skills (46 items)			T	P	S	0.95	0.97	0.91	0.97	0.96	0.95
Communication	Waiting one’s turn when speaking with other people	Respetar el turno al participar en conversaciones	7	7	6	0.84	0.78	0.66	0.86	0.77	0.79
Cooperation	Cooperating, doing what teachers say	Coopero, hago lo que los profesores dicen	6	6	7	0.91	0.85	0.75	0.90	0.85	0.81
Assertion	When someone considers they have not been well treated, express disagreement	Expresar desacuerdo al considerarse tratado injustamente	7	7	7	0.85	0.77	0.66	0.86	0.78	0.75
Responsibility	Doing what has to be done unprompted	Hago lo que debo sin que me lo tengan que decir	6	6	7	0.85	0.86	0.69	0.90	0.86	0.77
Empathy	Trying to understand other people’s feelings	Trato de pensar cómo se sienten los demás	6	6	6	0.93	0.85	0.70	0.91	0.87	0.82
Engagement	Trying to make new friends	Intento hacer nuevas amistades	7	7	7	0.89	0.87	0.75	0.88	0.85	0.76
Self-Control	Keeping calm when disagreeing with others	Mantengo la calma cuando estoy en descuerdo con los demás	7	7	6	0.89	0.84	0.82	0.91	0.84	0.81

Note: Nº items in Student form, Gresham et al. [9] Communication (7 items), Cooperation (6 items), Responsibility (6 items), Self-control (7 items).

**Table 3:** Correlations for teacher–parent (subscales and total).

Pearson Correlation ^a^, (Effect Size; Power %).								
	Teacher							
Parent	1	2	3	4	5	6	7	8
1. Communication	0.501 * (0.71; 92.61%)	0.314	0.183	0.479	0.302	0.440	0.248	0.463
2. Cooperation	0.582 * (0.76; 97.13%)	**0.367**	0.110	0.617 ** (0.78; 92.83%)	0.347	0.314	0.348	0.485 * (0.70; 91.34%)
3. Assertion	0.419	0.177	**0.272**	0.368	0.179	0.336	0.161	0.370
4. Responsibility	0.564 * (0.75; 96.40%)	0.302	0.285	0.507 * (0.71; 93.06%)	0.424	0.522 * (0.72; 94.08%)	0.229	0.543 * (0.74; 95.33%)
5. Empathy	0.659 ** (0.81; 95.89%)	0.336	0.018	0.553 ** (0.74; 95.85%)	**0.425**	0.344	0.459	0.507 * (0.71; 93.06%)
6. Engagement	0.425	0.104	0.346	0.204	0.316	0.516 * (0.71; 93.69%)	0.094	0.413
7. Self-control	0.287	−0.079	0.357	0.205	0.303	0.465	**−0.070**	0.312
8. Total SS	0.579 * (0.76; 97.01%)	0.253	0.276	0.495 * (0.70; 92.15%)	0.392	0.506 * (0.71; 92.98%)	0.240	0.525 * (0.72; 94.27%)

Notes: ^a^ *n* = 17. Scores for scales are standard scores. Scores for subscales are raw scores. SS = social skills. Boldface indicates convergent validity coefficients. * The correlation is significant at the 0.05 level (bilateral). ** The correlation is significant 0.01 (bilateral).

**Table 4:** Correlations for parent–student (subscales and total).

Pearson Correlations ^a^ (Effect Size; Power)								
	Student							
Parent	1	2	3	4	5	6	7	8
1. Communication	0.361 ** (0.60; 100%)	0.254 * (0.50; 99.97%)	0.164	0.228 * (0.48; 99.91%)	0.114	0.256 * (0.51; 99.97%)	0.160	0.308 ** (0.55; 99.98%)
2. Cooperation	0.314 ** (0.56; 99.98%)	0.275 ** (0.52; 99.90%)	0.128	0.331 ** (0.57; 100%)	0.276 ** (0.52; 99.90%)	0.148	0.380 ** (0.62; 100%)	0.378 ** (0.61; 100%)
3. Assertion	0.192	0.177	0.368 ** (0.61; 100%)	0.283 ** (0.49; 99.93%)	0.243 * (0.49; 99.95%)	0.372 ** (0.61; 100%)	0.134	0.376 ** (0.61; 100%)
4. Responsibility	0.365 ** (0.60; 100%)	0.328 ** (0.57; 100%)	0.266 ** (0.51; 99.86%)	0.311 ** (0.56; 99.98%)	0.298 ** (0.55; 99.96%)	0.248 * (0.5; 99.96%)	0.245 * (0.49; 99.95%)	0.420 ** (0.65; 100%)
5. Empathy	0.320 ** (0.57; 100%)	0.221 * (0.47; 99.87%)	0.152	0.208 * (0.46; 99.78%)	0.202 * (0.45; 99.72%)	0.127	0.186	0.282 ** (0.53; 99.93%)
6. Engagement	0.120	−0.015	0.396 ** (0.63; 100%)	0.054	0.084	0.366 ** (0.6; 100%)	−0.189	0.177
7. Self-control	0.406 ** (0.64; 100%)	0.284 ** (0.53; 99.93%)	0.194	0.281 ** (0.53; 99.93%)	0.168	0.264 ** (0.51; 99.85%)	0.297 ** (0.54; 99.96%)	0.386 ** (0.62; 100%)
8. Total SS	0.383 ** (0.62; 100%)	0.276 ** (0.52; 99.91%)	0.322 ** (0.57; 100%)	0.310 ** (0.57; 99.98)	0.253 * (0.50; 99.97%)	0.342 ** (0.49; 99.63%)	0.211 * (0.46; 99.81%)	0.431 ** (0.66; 100%)

Notes: ^a^ *n* = 98. SS = social skills. Boldface indicates convergent validity coefficients. * The correlation is significant at the 0.05 level (bilateral). ** The correlation is significant at the 0.01 level (bilateral).

**Table 5:** Standardized mean difference effect size for different between-raters’ scores on Total Social Skills Scale.

	MSS ^1^	SD ^1^	MSS ^2^	SD ^2^	|D|	ES
Male						
Parent–Teacher	99.61	13.98	99.66	15.20	0.06	0.00
Teacher–Student	99.66	15.20	96.30	15.97	3.37	−0.22
Parent–Student	99.61	13.98	96.30	15.97	3.31	−0.22
Female						
Parent–Teacher	100.57	16.53	100.46	14.91	0.10	0.01
Teacher–Student	100.46	14.91	103.00	13.76	2.54	0.18
Parent–Student	100.57	16.53	103.00	13.76	2.44	0.16

Notes: SS = social skills; |D| = absolute difference score; ES = effect size. ^1^ Mean standard score and standard deviation for first rater. ^2^ Mean standard score and standard deviation for second rater.

**Table 6:** Inter-rater agreement of Social Skills Scales for SESAS-SS scores.

	T-P		T-S		P-S	
	(*n* = 17)		(*n* = 88)		(*n* = 98)	
SESAS-SS scales	ICC	Sig.	ICC	Sig.	ICC	Sig.
	(Lower Bound–Upper bound)		(Lower Bound–Upper bound)		(Lower Bound–Upper bound)	
Social Skills	**0.493**	0.02	0.056	0.392	**0.604**	<0.001
	(−0.20–0.81)		(−0.43–0.38)		(0.41–0.73)	
Communication	**0.593**	0.03	0.221	0.125	**0.455**	<0.001
	(−0.02–0.85)		(−0.19–0.49)		(0.18–0.64)	
Cooperation	0.531	0.07	**0.4**	<0.001	**0.347**	0.003
	(−0.25–0.83)		(−0.04–0.64)		(0.01–0.57)	
Assertion	0.437	0.14	−0.17	0.770	**0.512**	<0.001
	(−0.66–0.80)		(−0.8–0.23)		(0.27–0.67)	
Responsibility	0.429	0.07	0.229	0.050	**0.382**	0.001
	(−0.25–0.77)		(−0.1–0.47)		(0.04–0.60)	
Empathy	**0.60**	0.04	−0.025	0.549	**0.333**	0.024
	(−0.10–0.86)		(−0.53–0.32)		(0.00–0.55)	
Engagement	**0.69**	0.01	−0.045	587	**0.526**	<0.001
	(0.11–0.89)		(−0.55–0.30)		(0.29–0.68)	
Self-Control	−0.07	0.61	0.17	0.116	**0.442**	0.001
	(−0.63–0.45)		(−0.15–0.42)		(0.18–0.62)	

Note: Two-way random-effects model (average) model. In boldface, the significant sample of ICC at the 0.05 bilateral level.

**Table 7:** Correlations for teacher–student (subscales and total).

Pearson Correlations ^a^ (Effect Size; Power)								
	Student							
Teacher	1	2	3	4	5	6	7	8
1. Communication	**0.169**	0.288 ** (0.54; 99.84%)	−0.047	0.077	−0.136	−0.068	−0.020	0.045
2. Cooperation	0.151	0.386 ** (0.62; 100%)	0.027	0.283 ** (0.53; 99.81%)	−0.186	−0.123	0.066	0.130
3. Assertion	−0.036	0.077	**−0.088**	−0.076	−0.076	0.012	−0.218 * (0.47; 99.67%)	−0.109
4. Responsibility	0.150	0.320 ** (0.57; 99.96%)	0.11	**0.185**	−0.188	−0.122	−0.016	0.063
5. Empathy	0.023	0.144	−0.048	−0.001	**−0.015**	−0.166	−0.188	−0.082
6. Engagement	0.033	0.180	0.004	0.089	0.009	**−0.024**	−0.120	0.022
7. Self-control	0.218 * (0.47; 99.67%)	0.411 ** (0.64; 100%)	−0.059	0.184	−0.111	−0.185	**0.128**	0.124
8. Total SS	0.119	0.307 ** (0.55; 99.93%)	−0.037	0.122	−0.119	−0.115	−0.066	**0.030**

Notes: ^a^ *n* = 98. SS = social skills. Boldface indicates convergent validity coefficients. * The correlation is significant at the 0.05 level (bilateral). ** The correlation is significant at the 0.01 level (bilateral).

**Table 8:** Comparison of social skills with stronger and weaker agreement across raters and studies.

Raters		Gresham et al., 2010 [9]	Gresham et al., 2018 [26]
Teacher–Parent			
Stronger	Social skills	Responsibility	Responsible decision-making
	ICC good agreement: Engagement, Empathy	Engagement	Self-management
	ICC fair agreement: Communication, Social skills		
Weaker	Self-control	Assertion Self-control	Self-awareness
Parent–Student			
Stronger	Social skills	Responsibility, Cooperation	Relationship skills
	ICC good agreement: Social skills		
	ICC fair agreement: Engagement, Assertion, Communication, Self-control		
Weaker	Empathy	Assertion Empathy	Self-awareness
	ICC poor agreement: Empathy, Cooperation, Responsibility		
Teacher–Student			
Stronger	Cooperation	Responsibility	Relationship skills
		Engagement	
	ICC fair agreement: Cooperation		
Weaker	-	Communication, Empathy	Self-awareness

## Data Availability

The data presented in this study are available on request from the corresponding author. The data are not publicly available due to the privacy of personal data.

## Supplementary Materials

The following supporting information can be downloaded at: https://www.mdpi.com/article/10.3390/bs12030062/s1, Mann-Whitney U Test; Table S1. Correlations Teacher-Parent (subscales and total, confidence interval included); Table S2. Correlations Parent-Student (subscales and total, confidence interval included) and Table S3. Correlations Teacher-Students (subscales and total, confidence interval included).
