# Supplementary material for: Multi-Informant Assessment of Adolescents’ Social–Emotional Skills: Patterns of Agreement and Discrepancy among Teachers, Parents, and Students
Source: Behav Sci (Basel). 2022 Feb 25;12(3):62. doi: 10.3390/bs12030062 (PMC8944948; doi:10.3390/bs12030062)
Supplement: Supplementary file 1 [file behavsci-12-00062-s001.zip › behavsci-1505065-supplementary.pdf]

behavioral sciences

MDPI

**Mann-Whitney U Test.**

To test whether males and females differ in terms of SS, a series of Mann-Whitney U Tests following Field [1] were performed (see supplementary materials). Female students returned values slightly, albeit significantly, higher than their male counterparts in the following scales and subscales: Total SS ($Mdn_{female} = 102.3$, $Mdn_{male} = 97.99$, $U = 194806.00$, $z = 6.72$, $p < 0.001$, $r = 0.2$) communication ($Mdn_{female} = 16$, $Mdn_{male} = 15$ $U = 195836.00$ , $z = 7$ , $p < 0.001$, $r = 0.21$) cooperation ($Mdn_{female} = 17$ , $Mdn_{male} = 15$ $U = 199193.50$, $z = 7.561$, $p < 0.001$, $r = 0.23$), assertion ($Mdn_{female} = 14$ $\bar{x} = 14.34$ , $Mdn_{male} = 14$ $\bar{x} = 13.35$ $U = 184317.50$, $z = 4.815$, $p < 0.001$, $r = 0.14$), responsibility ($Mdn_{female} =$ , $Mdn_{male} =$ $U = 203056.50$, $z = 8.28$, $p = 0.00$, $r = 0.25$), and empathy ($Mdn_{female} = Mdn_{male} =$ $U = 200918.00$, $z = 7.91$, $p < 0.001$, $r = 0.24$). The effect sizes of these differences in the subscales (Cohen, 1988; Sawilowsky, 2009) were small (ranging from 0.14 -assertion- to 0.25 -responsibility). No significant differences were found between the genders in engagement ($Mdn_{female} = Mdn_{male} = 17$, $U = 162931.00$, $z = 0.87$, $p = 0.38$) or self-control ($Mdn_{female} = Mdn_{male} = 12$, $U = 157711.00$, $z = -.09$, $p = 0.93$). No significant differences were found either among teachers and parents regarding their estimates of SS according to the students' gender in total SS or in communication, cooperation, assertion, responsibility, empathy, engagement, and self-control (Teachers: total SS $U = 966.00$, $z = 0.19$, $p = 0.85$; communication, $U = 838.50$, $z = -.89$, $p = 0.37$; cooperation, $U = 1028.5$, $z = 0.72$, $p = 0.47$; assertion, $U = 845.50$, $z = -.83$, $p = 0.41$; responsibility, $= 1000.50$, $z = 0.48$, $p = 0.63$; empathy, $U = 1109.00$, $z = 1.41$, $p = 0.16$; engagement, $U = 1105.50$, $z = 0.53$ , $p = 0.6$, and self-control $U = 922.00$, z = -.18, p = 0.85. Parents: U = 1243.00, z = 0.6, p = 0.55; communication, U = 1255.50, z = 0.69, $p = 0.49$; cooperation, $U = 1389.00$, $z = 1.7$, $p = 0.09$; assertion, $U = 1192.50$, $z = 0.24$, $p = 0.81$; responsibility, $U = 1327.50$, $z = 1.22$, $p = 0.22$; empathy, $U = 1243.50$, $z = 0.61$, $p = 0.54$; engagement, $U = 1064.00$, $z = -.70$, $p = 0.49$, and self-control, $U = 1258.00$, $z = 0.71$, $p = 0.48$)

**Table S1.** Correlations Teacher-Parent (subscales and total, confidence interval included).

*Pearson Correlation*[a]
(95% Confidence Interval)
(Effect Size; Power %)

| | Teacher | | | | | | | |
|---|---|---|---|---|---|---|---|---|
| **Parent** | **1** | **2** | **3** | **4** | **5** | **6** | **7** | **8** |
| 1. Communication | **.501*** (.03-.79) (.71; 92.61%) | .314 (-.2-.69) | .183 (-.33-61) | .479 (-.00-.78) | .302 (-.21-.68) | .440 (-.05-.76) | .248 (-.26-.65) | .463 (-.02-.77) |
| 2. Cooperation | .582* (.14-.83) (.76; 97.13%) | **.367** (-14-.72) | .110 (-.39-.56) | .617** (.19-.85) (.78; 92.83%) | .347 (-.16-.71) | .314 (-.2-.69) | .348 (-.16-.71) | .485* (.01-.78) (.70; 91.34%) |
| 3. Assertion | .419 (-.08-.75) | .177 (-33-.61) | **.272** (-.24-.67) | .368 (-.14-.72) | .179 (-.33-.61) | .336 (-.17-.7) | .161 (-35-.59) | .370 (-.13-.72) |
| 4. Responsibility | .564* (.11-.82) (.75; 96.40%) | .302 (-.21-.68) | .285 (-.23-.67) | **.507*** (.03-.79) (.71; 93.06%) | .424 (-.07-.75) | .522* (.06-.8) (.72; 94.08%) | .229 (-.28-.64) | .543* (-.13-.81) (.74; 95.33%) |
| 5. Empathy | .659** (.26-.86) (.81; 95.89%) | .336 (-.17-.7) | .018 (-.47-.49) | .553* (.1-.82) (.74; 95.85%) | **.425** (-.07-.75) | .344 (-.16-.71) | .459 (-.03-.77) | .507* (.03-.79) (.71; 93.06%) |
| 6. Engagement | .425 (-.07-.75) | .104 (-.4-.56) | .346 (-.16-.7) | .204 (-.31-.62) | .316 (-.19-.69) | **.516*** (.05-.8) (.71; 93.69%) | .094 (-.4-.55) | .413 (-.08-.75) |
| 7. Self-control | .287 (-.22-.67) | -.079 (-.54-.42) | .357 (-.15-.71) | .205 (-.31-62) | .303 (-.21-.68) | .465 (-.02-.8) | **-.070** (-.53-.42) | .312 (-.2-.69) |
| 8. Total SS | .579* (.13-.83) (.76; 97.01%) | .253 (-.26-.65) | .276 (-.24-.67) | .495* (.02-79) (.70; 92.15%) | .392 (-.11-.73) | .506* (.03-.79) (.71; 92.98%) | .240 (-.27-.65) | **.525*** (.06-.80) (.72; 94.27%) |

Notes: [a] $n = 17$. Scores for scales are standard scores. Scores for subscales are raw scores. SS = social skills. Boldface indicates convergent validity coefficients. *. The correlation is significant at the 0.05 level (bilateral). **. The correlation is significant 0.01 (bilateral).

**Table S2.** Correlations Parent-Student (subscales and total, confidence interval included).

| | ***Pearson Correlations***[a] **(95% Confidence Interval) (Effect Size; Power)** | | | | | | | |
|---|---|---|---|---|---|---|---|---|
| | **Student** | | | | | | | |
| **Parent** | **1** | **2** | **3** | **4** | **5** | **6** | **7** | **8** |
| 1. Communication | **.361**** (.17-.52) (.60; 100%) | .254* (.06-.43) (.50; 99.97%) | .164 (-.04-.35) | .228* (.03-.41) (.48; 99.91%) | .114 (-.09-.31) | .256* (.06-.43) (.51; 99.97%) | .160 (-.04-.35) | .308** (.12-.48) (.55; 99.98%) |

| | | | | | | | | |
|---|---|---|---|---|---|---|---|---|
| 2. Cooperation | .314$^{**}$<br>(.12-.48)<br>(.56; 99.98%) | **.275**$^{**}$<br>(.08-.45)<br>(.52; 99.90%) | .128<br>(-.07-.32) | .331$^{**}$<br>(.14-.5)<br>(.57; 100%) | .276$^{**}$<br>(.08-.45)<br>(.52; 99.90%) | .148<br>(-.05-.34) | .380$^{**}$<br>(.2-.54)<br>(.62; 100%) | .378$^{**}$<br>(.2-.54)<br>(.61; 100%) |
| 3. Assertion | .192<br>(-.01-.38) | .177<br>(-.02-.36) | **.368**$^{**}$<br>(.18-.53)<br>(.61; 100%) | .283$^{**}$<br>(.09-.46)<br>(.49; 99.93%) | .243$^{*}$<br>(.05-.42)<br>(.49; 99.95%) | .372$^{**}$<br>(.19-.53)<br>(.61; 100%) | .134<br>(-.07-.32) | .376$^{**}$<br>(.19-.53)<br>(.61; 100%) |
| 4. Responsibility | .365$^{**}$<br>(.18-.52)<br>(.60; 100%) | .328$^{**}$<br>(.14-.49)<br>(.57; 100%) | .266$^{**}$<br>(07-.44)<br>(.51; 99.86%) | **.311**$^{**}$<br>(.12-.48)<br>(.56; 99.98%) | .298$^{**}$<br>(11-.17)<br>(.55; 99.96%) | .248$^{*}$<br>(.05-.43)<br>(.5; 99.96%) | .245$^{*}$<br>(.05-.42)<br>(.49; 99.95%) | .420$^{**}$<br>(.24-.47)<br>(.65; 100%) |
| 5. Empathy | .320$^{**}$<br>(.13-.49)<br>(.57; 100%) | .221$^{*}$<br>(.02-.4)<br>(.47; 99.87%) | .152<br>(-.05-.34) | .208$^{*}$<br>(.01-.39)<br>(.46; 99.78%) | **.202**$^{*}$<br>(.00-.38)<br>(.45; 99.72%) | .127<br>(-.07-.32) | .186<br>(-.01-.37) | .282$^{**}$<br>(.09-.45)<br>(.53; 99.93%) |
| 6. Engagement | .120<br>(-.08-.31) | -.015<br>(-.21-.18) | .396$^{**}$<br>(.21-.55)<br>(.63; 100%) | .054<br>(-.15-.25) | .084<br>(-.12-.28) | **.366**$^{**}$<br>(.18-.53)<br>(.6; 100%) | -.189<br>(-.37-.01) | .177<br>(-.02-.36) |
| 7. Self-control | .406$^{**}$<br>(.23-.56)<br>(.64; 100%) | .284$^{**}$<br>(.09-.46)<br>(.53; 99.93%) | .194<br>(-.00-.38) | .281$^{**}$<br>(.09-.54)<br>(.53; 99.93%) | .168<br>(-.03-.35) | .264$^{**}$<br>(.07-.44)<br>(.51; 99.85%) | **.297**$^{**}$<br>(.10-.47)<br>(.54; 99.96%) | .386$^{**}$<br>(.20-.54)<br>(.62; 100%) |
| 8. Total SS | .383$^{**}$<br>(.2-.54)<br>(.62; 100%) | .276$^{**}$<br>(.08-.45)<br>(.52; 99.91%) | .322$^{**}$<br>(.13-.5)<br>(.57; 100%) | .310$^{**}$<br>(.12-.48)<br>(.57; 99.98) | .253$^{*}$<br>(.06-.43)<br>(.50; 99.97%) | .342$^{**}$<br>(.15-.51)<br>(.49; 99.63%) | .211$^{*}$<br>(.01-.39)<br>(.46; 99.81%) | **.431**$^{**}$<br>(.25-.58)<br>(.66; 100%) |

Notes: $^{a}$ $n$ = 98. SS = social skills. Boldface indicates convergent validity coefficients. *. The correlation is significant at the 0.05 level (bilateral). **. The correlation is significant at the 0.01 level (bilateral).

**Table S3.** Correlations Teacher -Students (subscales and total, confidence interval included).

*Pearson Correlations*[a]

(95% Confidence Interval)

(Effect Size; Power)

| | Student | | | | | | | |
|---|---|---|---|---|---|---|---|---|
| Teacher | 1 | 2 | 3 | 4 | 5 | 6 | 7 | 8 |
| 1. Communication | **.169**<br>(-.04-.37) | .288**<br>(.08-.47)<br>(.54; 99.84%) | -.047<br>(-.25-.16) | .077<br>(-13-.28) | -.136<br>(.34-.08) | -.068<br>(-.27-.14) | -.020<br>(.23-.19) | .045<br>(-.17-.25) |
| 2. Cooperation | .151<br>(-.06-.35) | **.386****<br>(.19-.55)<br>(.62; 100%) | .027<br>(-.18-.23) | .283**<br>(.08-.46)<br>(.53; 99.81%) | -.186<br>(-.38-.02) | -.123<br>(-.32-.09) | .066<br>(-14-.27) | .130<br>(-.08-.33) |
| 3. Assertion | -.036<br>(-.24-.17) | .077<br>(-.13-.28) | **-.088**<br>(-.29-.12) | -.076<br>(-.28-.14) | -.076<br>(-.28-.13) | .012<br>(-19-.22) | -.218*<br>(-.41--.01)<br>(.47; 99.67%) | -.109<br>(-.31-.10) |
| 4. Responsibility | .150<br>(-.06-.35) | .320**<br>(.12-.5)<br>(.57; 99.96%) | 011<br>(-.2-.22) | **.185**<br>(-.03-.38) | -.188<br>(-.38-.02) | -.122<br>(-.32-.09) | -.016<br>(-.22-.19) | .063<br>(-.15-.27) |
| 5. Empathy | .023<br>(-.19-.23) | .144<br>(-.07-.34) | -.048<br>(-.25-.16) | -.001<br>(-.21-.21) | **-.015**<br>(-.22-.19) | -.166<br>(-.36-.04) | -.188<br>(-.38-.02) | -.082<br>(-.29-.13) |
| 6. Engagement | .033<br>(-.18-.24) | .180<br>(-.03-.37) | .004<br>(-.21-.21) | .089<br>(-.12-.29) | .009<br>(-.20-.22) | **-.024**<br>(-.23-.19) | -.120<br>(-.32-.09) | .022<br>(-.19-.23) |
| 7. Self-control | .218*<br>(.01-.41)<br>(.47; 99.67%) | .411**<br>(.22-.57)<br>(.64; 100%) | -.059<br>(-.26-.15) | .184<br>(-.03-.38) | -.111<br>(-.32-.1) | -.185<br>(-.38-.02) | **.128**<br>(-.08-.33) | .124<br>(-.09-.32) |
| 8. Total SS | .119<br>(-.09-.32) | .307**<br>(.01-.48)<br>(.55; 99.93%) | -.037<br>(-.24-.17) | .122<br>(-.09-.32) | -.119<br>(-.32-.09) | -.115<br>(-.32-.1) | -.066<br>(-.27-.14) | **.030**<br>(-.18-.24) |

Notes: [a] $n$ = 98. SS = social skills. Boldface indicates convergent validity coefficients. *. The correlation is significant at the 0.05 level (bilateral). **. The correlation is significant at the 0.01 level (bilateral).

## References

[1] Field, A. *Discovering Statistics Using IBM SPSS Statistics*, 5 ed.; Sage Publications Ltd, U.K.: 2018
